# Women’s Health Across the Lifespan: A Sex- and Gender-Focused Perspective

**DOI:** 10.1093/ptj/pzae121

**Published:** 2024-08-31

**Authors:** Jessica L McKinney, Susan C Clinton, Laura E Keyser

**Affiliations:** Mama LLC, Canton, Massachusetts, USA; LTI Physio LLC, Sault Sainte Marie, Michigan, USA; Mama LLC, Canton, Massachusetts, USA; Department of Physical Therapy and Rehabilitation Science, University of California, San Francisco, California, USA

**Keywords:** Female, Health Disparities, Life Course Health Development, Sex and Gender, Women’s Health

## Abstract

Women’s health in physical therapy has historically focused on sexual and reproductive health. The biological and social constructs of sex and gender, respectively, are determinants of health, including pathophysiology of disease and therapeutic outcomes, and an expansion of the concept of “women’s health” is warranted. This Perspective explores the role of sex and gender as key determinants of women’s and girls’ health and highlights the factors pertinent to physical therapist practice. The Scale for the Assessment for Narrative Review Articles (SANRA), a 6-point assessment to evaluate the quality of narrative reviews, was used a priori and consulted throughout. Across the lifespan, sex- and gender-based health disparities exist. These include sex-based disparities in maternal–fetal outcomes linking female fetal sex to maternal hypertensive disorders of pregnancy, along with a sex-based female advantage in birth outcomes and the emergence of gender differences in motor development. A complex interplay of biological and socially influenced factors contributes to an increased care burden for women throughout adulthood and specific risks for the development of cardiovascular and pelvic floor conditions, decreased function, and increased disability. Sex- and gender-disaggregated data are lacking in outcomes literature. A sex- and gender-informed approach in physical therapy, including analyzing data by sex and gender, may better meet the needs of patients and better prepare physical therapist professionals to contribute to women’s health across the lifespan. Success will take coordinated effort involving many stakeholders within and adjacent to the physical therapist community. The influence of sex and gender are lifelong determinants of health, making them critically important to consider in physical therapist practice, education, research, advocacy, and policy. In women’s health, focusing on sexual and reproductive health is limiting and insufficient.

## Introduction

Contemporary language characterizes women’s health as including health conditions and experiences unique to women, along with those experienced differently or disproportionately by women, representing an expansion of historical precedent.^1^

The following definitions of “Women” and “Women’s Health” apply to this article^1^:

“Women: When using the term women in the context of “women’s health,” “women” is inclusive of both sex as a biological variable and gender as a social variable across the life course, including girls and adolescents. This includes people assigned female at birth, transgender women, transgender men, and nonbinary people affected by the topics covered by this report. The authors recognize that not all people who identify as women have the same reproductive anatomy and not all people who were assigned female at birth identify as women.

Women’s Health: The phrase “women’s health” is used to highlight the interest in all areas of health related to women, including conditions associated with both sex as a biological variable and gender as an intersecting social determinant of health. This includes diseases and conditions that present only in women, disproportionately in women, and differently in women.”

In physical therapy, as in the rest of health care, “women’s health” has been actualized through a near-exclusive focus on sexual and reproductive health (SRH) and has otherwise adopted a gender-blind approach to caring for women and girls. Gender-blindness is “nonawareness of the fact that a great deal of knowledge is based on research performed with men and males thus fostering a neutralization across sex and gender.”^2^^,^^3^

**Figure 1:**
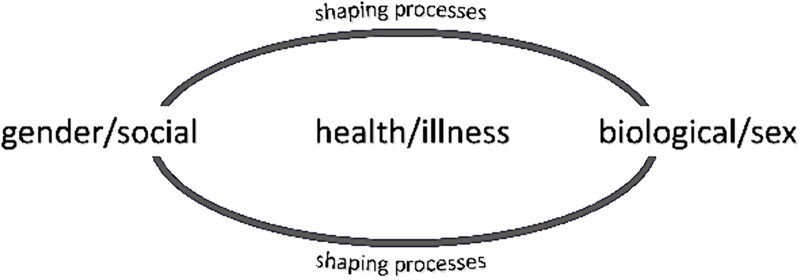
Shaping processes of the gender-biology nexus. Reprinted with permission from Annandale E, Wiklund M, Hammarström A. Theorising women’s health and health inequalities: shaping processes of the “gender-biology nexus.” *Glob Health Action*. 2018;11(sup3):1669353. doi: 10.1080/16549716.2019.1669353.

Sex is a biological construct based on differences in anatomy, genes, hormones, and physiology. Sex is typically described dichotomously (male and female), though also includes intersex.^4^ Gender refers to “socially constructed norms that impose and determine roles, relationships, and positional power for all people across their lifetime.”^5^ Man and woman are gender designations; however, gender is not binary, and gender inclusivity affords additional descriptors. Though distinctly defined, sex and gender have a “shaping and reinforcing effect” on each other.^6^ Annandale et al describe an “imbricated and recursive process whereby the gendered experiences influence biology, and biology is adaptive and remade in response to its environment.”^6^ Examples include (1) societally influenced body standards for women that contribute to higher rates of dieting, decreased physical labor, and higher rates of osteoporosis (the gender-shaping of biology) and (2) a woman’s pregnancy influencing workplace role and advancement due to perceptions of workplace decision-makers (biologic-shaping of gender). Figure 1 depicts how this influences health and thus is relevant to health care professionals.^6^

Male cells, mice, and humans comprise the primary research participants informing much of what is known about health and disease. Perceived greater biological complexity (eg, female reproductive hormones) and potential risks to females and their offspring rendered them as less suitable participants for research. In 1993, the United States (US) National Institutes of Health (NIH) mandated inclusion of females in NIH-funded clinical trials with human participants.^7^ This led to greater numbers of females included in trials. However, the impact of the policy was limited by failures to disaggregate and analyze trial data by sex.^8^

This problem is not unique to medicine and basic science research. As an example, in 2020, Cieza et al estimated the global need for rehabilitation, finding that approximately one in 3 people require rehabilitation during the course of an injury or illness.^9^ The authors described prevalence estimates and years lost to disability for men and women and noted greater burden among women. Musculoskeletal issues contributed most to global rehabilitation needs for men and women with lower back pain (LBP) cited as the most burdensome health condition. However, among 11 Cochrane Libraries systematic reviews supporting effectiveness of rehabilitation for LBP, outcomes were not disaggregated by sex or gender except for the review exclusive to pregnancy, thus eliminating the possibility of understanding if rehabilitation outcomes for LBP may differ by sex or gender.^9^

Since the introduction of the term gender in the 1970s as distinct from sex, the terms have been often conflated in biomedical research and education, frequently using women and female as equivalent and interchangeable.^6^ Furthermore, females and women have had their physiology and health measured against males and men as the norm to which all things human default.^4^^,^^6^ Respectively, sex and gender are biological and social determinants of health. Recent publications draw attention to relevant health differences and identify areas for future study.^4^^,^^8^

Gender norms are culturally and societally defined traits and classically express male power dominance. According to prevailing gender norms, men and women are considered inherently different, and male traits are valued over female traits.^3^ Health care leadership has the same gender bias, with authors highlighting lack of equity in research, teaching, and clinical leadership positions and increasingly calling for this to be addressed.^10^ Within health care delivery, gender norms may influence the occurrence of medically unmotivated treatment differences disadvantaging women.^11^^,^^12^ Within the physical therapist community, Bisconti et al identified a need for gender medicine training.^13^ As women live longer than men, experience a greater burden of disability, and commonly present in all spheres of physical therapist practice, it is important for the physical therapy community to be better equipped to care for women and girls across the lifespan, including but not limited to SRH.

**Figure 2:**
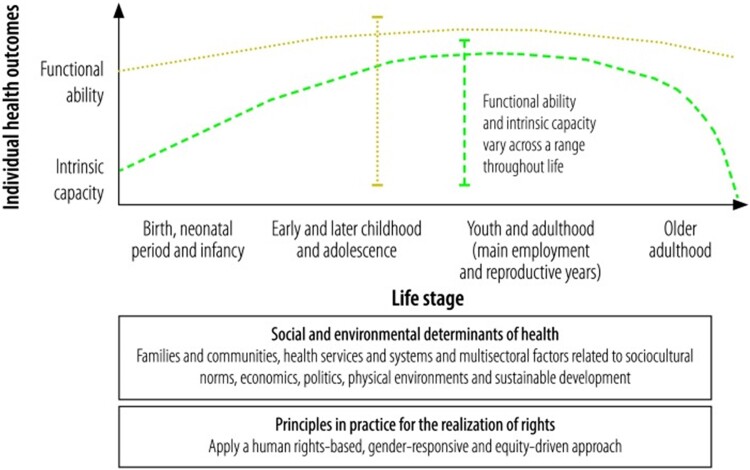
A conceptual framework for life course health development. This graphic represents the relationship between intrinsic capacity and functional ability across the lifespan, influenced by social, environmental, and political determinants of health. Reprinted with permission from Kuruvilla S, Sadana R, Montesinos EV, et al. A life-course approach to health: synergy with sustainable development goals. *Bull World Health Organ*. 2018;96:42–50. doi: 10.2471/BLT.17.198358.

The objective of this narrative review is to explore the role of sex and gender as key determinants of women’s and girls’ health and to highlight factors pertinent to physical therapist practice and to the health of women and girls, utilizing principles of Life Course Health Development (Fig. 2) to organize key findings according to life stage. It purposely moves away from an SRH-focused interpretation of “Women’s Health Physical Therapy” to one that includes SRH, but also recognizes that physical therapists care for female patients across the lifespan and benefit from integrating these concepts of women and girls, health, and physical therapy simultaneously.^14^ This research focuses on female, girls', and women’s health in a binary sex and gender capacity, an approach undertaken as an academic starting point, not a finish line, in expanding women’s health. Where possible, gender- and sex-specific terms are used, especially where these reflect cited literature. However, given the ubiquity with which terms are used interchangeably (eg, female and woman) in research and policy, they will occasionally be used interchangeably in this manuscript.

## Narrative Review Search

In writing this perspective, we appraised a wide body of evidence, to synthesize findings and generate key insights. Narrative reviews are regarded as influential and a core component of health care literature. The Scale for the Assessment for Narrative Review Articles (SANRA) is a critical appraisal and quality assessment tool for narrative reviews (Suppl. Material 1).^15^ The SANRA was used a priori and consulted throughout the review process to ensure the highest rating for each item was met. According to SANRA guidelines, this narrative review was justified as important for the readership because the physical therapist profession participates in clinical care and research involving women and no similar review could be identified summarizing women’s health through a sex- and gender-based lens for the physical therapy profession, though similar articles have been published in the medical literature. This informed the development of the research aim to explore the role of sex and gender as key determinants of women’s and girls’ health and to highlight factors pertinent to physical therapist practice and to the health of women and girls. Literature was evaluated and included according to the authors’ judgement of applicability to the physical therapist profession. The full list of resources on topics addressed in the manuscript can be found in Supplementary Material 2.

PubMed was searched for publications in English published on or before January 31, 2022 using “sex” or “gender,” or “women’s health” and “rehabilitation,” “physical therapy,” or “physiotherapy.” The search included these terms in combination with health conditions of interest. PubMed “Similar Articles,” and “Cited By” features were used to expand the search to identify seminal and/or representative articles. The literature search was enhanced by reviewing reference lists of identified articles and retrieving relevant citations. This was supplemented by searches of GoogleScholar, *Journal of Women’s Health Physical Therapy* archives and websites/publications of the United Nations, World Health Organization, Centers for Disease Control, NIH, and similar governmental and nongovernmental organizations with resources relevant to women’s health. Influential original studies were selected for inclusion; however, due to space limitation and the breadth of the subject matter, representative reviews were frequently included.

Results of the literature search reflective of sex- and gender-based differences are presented and are grouped by the following 5 life stages: (1) maternal and fetal development and influence, (2) infancy and childhood, (3) adolescence and young adulthood, (4) middle-late adulthood, and (5) older adulthood. Within each stage and to the degree data were identified, known health disparities, the influencing role of sex and gender on health, and reported implications are presented.

## Maternal and Fetal Development and Influences

Every cell in the body is sex-specific, and evidence suggests sex-based differences in maternal and neonatal health outcomes according to fetal sex.^16^^,^^17^ Hypertensive disorders of pregnancy (HDP) include gestational hypertension, preeclampsia, and eclampsia and significantly contribute to maternal morbidity and mortality. Female fetal sex has been identified as a risk factor for gestational hypertension and preeclampsia, whereas male fetal sex has been identified as an independent risk factor for preterm delivery, failure to progress during first- and second-stage labor, and cesarean section.^18–20^ In a large study comparing 53,104 pregnancies with a female fetus to 55,891 with a male fetus, male fetuses had higher rates of macrosomia, which can negatively influence labor progression and need for cesarean section.^19^ Moreover, both male and female fetal sex appear to differently influence diabetes risk, with pregnancies of male fetal sex having increased risk of gestational diabetes, and pregnancies with female sex increasing the risk of type 2 diabetes mellitus after pregnancy.^19^^,^^21^^,^^22^

While fetal sex influences maternal health, maternal health influences neonatal health in sex-specific ways. Females have a survival advantage, living longer than males even in harsh conditions.^23^ This is observed in infancy, where females have better survival and health outcomes, particularly after preterm delivery.^24^ One explanation is that the placenta adapts in sex-specific ways, protective to female fetuses.^25^ Additionally, maternal overweight or obesity has been linked to adverse maternal, neonatal, and child health outcomes. However, a study of 955 mother–infant pairs found the risk of obesity in children at 1-year existed only in male offspring.^26^ Similarly, a study of the influence of intrauterine exposure to gestational diabetes found that for males, not females, this was a risk factor for childhood obesity at 5 to 7 years old.^27^ This complex biological interaction is further mediated by maternal stress, which can be heavily influenced by social factors, including gender (gender roles, gender norms), race or racism, socioeconomic factors, among others.^14^

## Infancy and Childhood

Gender bias exists at all life stages and has been documented in infancy. In a study of motor development of 11-month-old male and female infants, investigators found no difference in motor performance between groups, though mothers of male infants overestimated infants’ performance, and mothers of female infants underestimated their ability.^28^ Reinforcing this finding, a large multinational study of children 4 to 24 months found no gender-based differences in major motor milestones.^29^ At these early stages, inherent sex and gender differences appear absent or minimal. However, infants and young children are highly responsive to social and environmental cues, including gender norms and expectations. In a study of 6- to 9-month-old infants, females demonstrated higher fine motor skills during independent play, and males demonstrated higher intensity play scenarios.^30^ Researchers observed that parents’ statements differentially promoted gross motor skills in males and fine motor skills in females; however, parents perceived that other adults promoted gender differences, but they did not.

Motor performance in childhood differs between boys and girls, with multiple studies finding that boys exhibit an advantage in gross motor skills.^31^^,^^32^ Gender differences in physical activity (PA) exist among preschool-age children, whereby boys engage in more moderate–vigorous PA and less sedentary behavior than girls.^33^ PA has many health benefits and offers one strategy for healthy weight maintenance and weight loss in the context of increasing prevalence of childhood overweight and obesity. Girls (compared to boys) and children with obesity (compared to normal weight) are less likely to meet recommendations for age-appropriate PA.^34^

## Adolescence and Young Adulthood

Physical fitness of children and adolescents has declined globally, with a significant negative trend for cardiorespiratory fitness.^35^ A positive association between cardiorespiratory fitness and mental health has been observed in older adolescents and is strongest for girls, suggesting the value of PA among prepubertal and adolescent girls.^36^ In work comparing BMI-matched older adolescents who were active (**≥**60 minutes of vigorous PA per day) versus those who were sedentary, significant differences were identified in circulating inflammatory mediators, growth factors, fitness, and bone mineralization, with all findings favoring the vigorous PA group.^37^ As evidence grows to support the claim that adult diseases have origins in childhood health and lifestyle patterns, the need for targeted health promotion is reinforced.^14^ Health promoting efforts in adolescence must account for gender disparities in physical self-perception and self-reported overall physical fitness, both of which demonstrate that girls are disadvantaged. Among 12- to 17-year-old adolescents, girls reported negative body attractiveness, sport competence, physical strength, and self-confidence, which significantly influenced negative self-reported overall physical fitness.^38^

**Figure 3:**
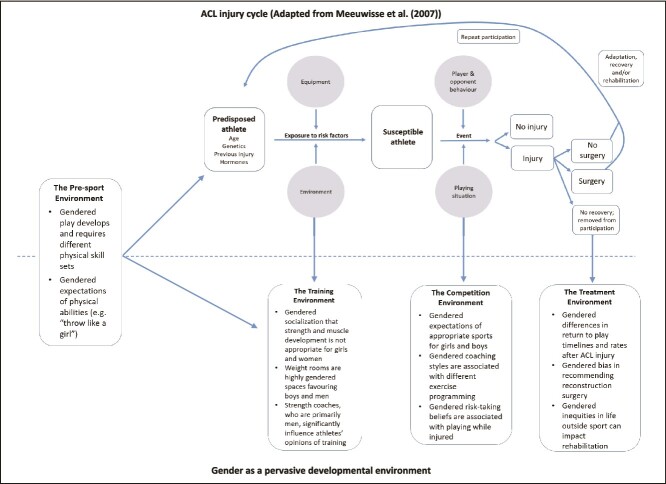
Gender as a pervasive developmental environment in the ACL injury cycle. ACL = anterior cruciate ligament. Reprinted with permission from Parsons JL, Coen SE, Bekker S. Anterior cruciate ligament injury: towards a gendered environmental approach. *Br J Sports Med*. 2021;55:984–990. doi: 10.1136/bjsports-2020-103,173.

**Figure 4:**
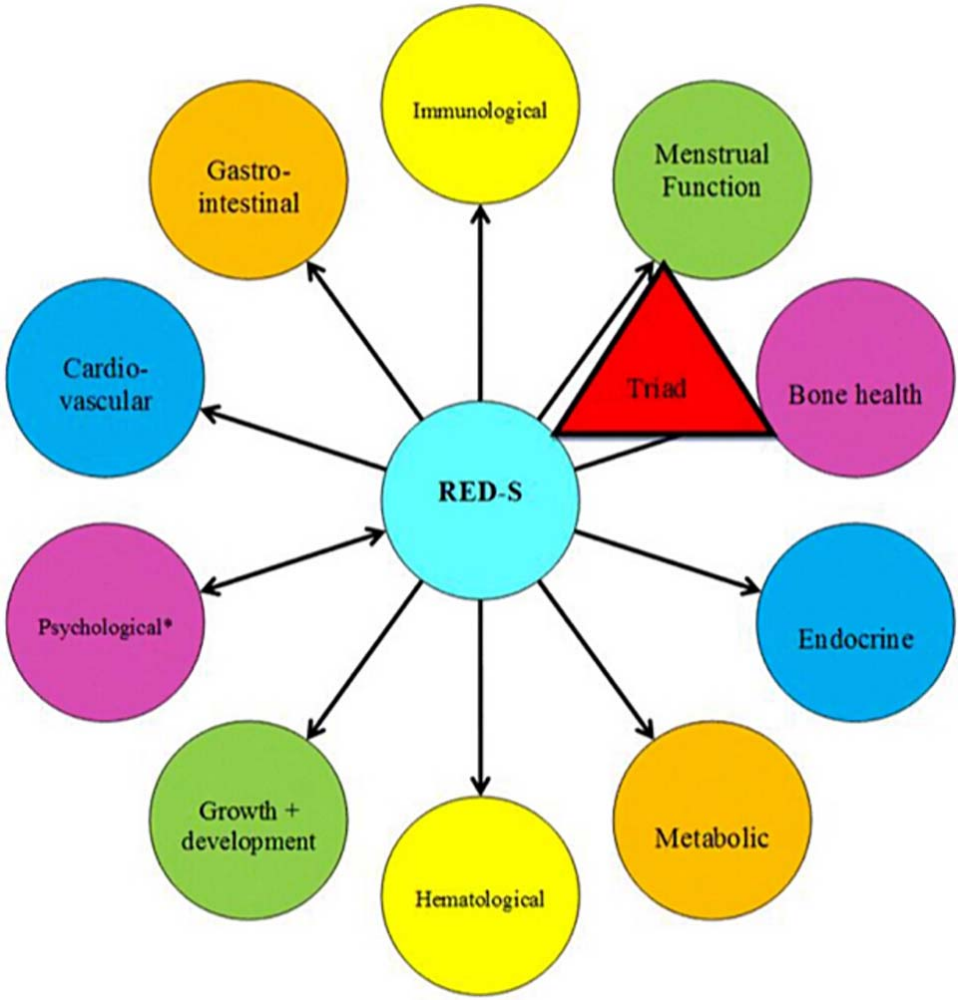
Health consequences of Relative Energy Deficiency in Sport (RED-S). Reprinted with permission from Mountjoy M, Sundgot-Borgen JK, Burke LM, et al. IOC consensus statement on relative energy deficiency in sport (RED-S): 2018 update. *Br J Sports Med*. 2018;52:687–697. doi: 10.1136/bjsports-2018-099193.

Evidence suggests that sex and gender differences in motor performance during childhood persist in adolescence and that enhanced motor learning among males during the adolescent period may account for some of these observed differences.^38^^,^^39^ What remains to be understood is the extent to which, if at all, these are inherent sex-based differences or are the result of boys growing up in an environment that expects and fosters higher motor skills, in comparison with the expectation and fostering of linguistic and fine motor skills in girls. In their review, Parsons et al highlight gendered environmental influences as a factor in the 3- to 6-fold risk of anterior cruciate ligament (ACL) injury experienced by girls and women compared to boys and men.^40^ For over 20 years, researchers and clinicians have emphasized sex-based interpretations of risk factors (eg, Q-angle, structural knee valgus, menstrual phase, etc.) in prevention and treatment programs without yielding a decrease in rate of ACL injury. The authors argue that gendered environments, not intrinsic and extrinsic risk factors of individual bodies, require study and action. Their proposed model (Fig. 3) demonstrates the consideration of “gender as a pervasive developmental environment,” depicting how this influences “presport, training, and competition environments through to ACL injury and the treatment environment.”^40^ While the authors developed this model for ACL injury and rehabilitation, it may prove useful for researchers and clinicians working with female athletes broadly. The Female Athlete Triad, now called Relative Energy Deficiency in Sport (RED-S) represents another example of gendered influences on female biology (Fig. 4).^41^ Though boys and men can experience RED-S, the condition disproportionately affects girls and women, who are more likely to experience disordered or restrictive eating. The impacts of low energy availability on bone health, menstrual, metabolic, gastrointestinal, and endocrine function are pronounced and may have lifelong effects on a women’s health.^41^

## Middle-Late Adulthood

Women act as **“**shock absorbers**”** in society in normal times and in times of crisis.^42^ This has recently been demonstrated in the numbers of women who lost paid employment or left the workforce due to the disproportionate caregiving burden that befell them during the COVID-19 pandemic.^43^ Crises aside, women contribute the majority of paid and unpaid caregiving, including child and elder care. Unpaid/informal caregiving is linked to negative health and economic consequences for women and for society, at large.^44^ This manifests as missed earning opportunities and deprioritization of personal health through decreased health habits (eg, PA, healthy eating), procrastination to address personal health issues, and impaired ability to fulfill treatment recommendations. One of the earliest drivers of this gendered caregiving burden is the transition to parenthood. Paid maternity leave significantly predicts lower odds of maternal and infant re-hospitalization and higher odds of favorable stress management and exercise, yet the United States lacks federal policy guaranteeing paid parental leave.^45^

**Figure 5:**
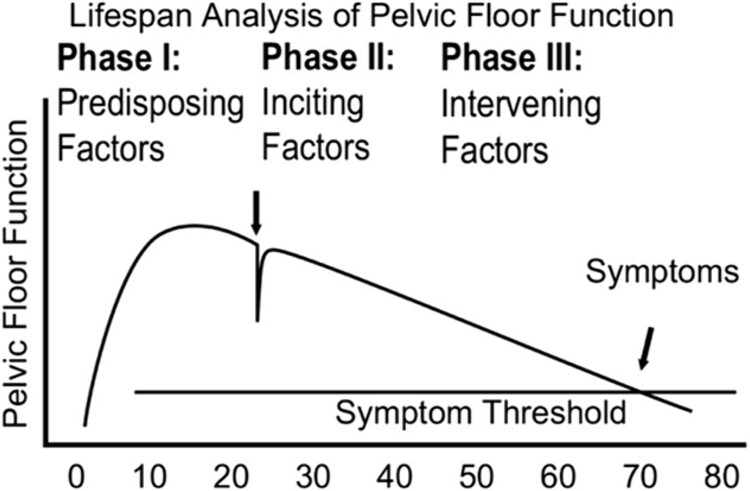
Integrated lifespan analysis of pelvic floor function. This graphical display of the abstract concept of pelvic floor function tracks the functional reserve throughout different phases of a woman’s lifespan. Initially, pelvic floor structure growth in late teens leads to a fully developed pelvic floor. Vaginal birth affects pelvic floor function. Finally, age-related deterioration occurs until a symptom threshold is reached where the functional reserve present earlier in life is lost. Reprinted with permission from Delancey, John O. L., Kane Low, L., Miller, Janis M., Patel, D.A. and Tumbarello J. A Graphic integration of causal factors of pelvic floor disorders: an integrated lifespan model. *Am J Obstet Gynecol*. 2008;199:1–12. doi: 10.1016/j.ajog.2008.04.001.Graphic.

Pregnancy and childbirth represent seminal health events for a woman’s current and future health. Two related conditions important to rehabilitation professionals are pelvic floor disorders (PFDs) and HDP. PFDs include urinary incontinence (UI), fecal incontinence, and pelvic organ prolapse and affect at least 25% of women in the United States.^46^ Pregnancy and childbirth are major risk factors for PFDs.^46^^,^^47^ Following spontaneous vaginal childbirth, peak incidence of urinary and fecal incontinence occurs at 5 to 7 years and approximately 15 years for pelvic organ prolapse.^47^ A lifespan model for understanding pelvic floor function describes pelvic floor functional reserve as something that is maximized in the early decades of life and prior to pregnancy and childbirth (Fig. 5).^48^ Inciting factors will challenge functional reserve (eg, pregnancy, childbirth) and intervening factors may negatively or positively influence functional reserve (eg, obesity, aging, pelvic floor muscle training, functional mobility).

HDP affects 8% to 10% of pregnancies, and rates are rising, due to increasing maternal age and increased negative health markers, such as overweight, obesity, hypertension, diabetes, or impaired glucose tolerance.^49^ Women with HDP experience lifelong increased cardiac risk, even when normotensive in the postpartum period. HDP and related complications (gestational diabetes, preterm delivery) represent independent risk factors for cardiovascular mortality and morbidity.^50^ Preeclampsia conveys a relative risk for chronic hypertension of 3.7 (95% CI = 2.70–5.05) and risk ratios of 4.19 (95% CI = 2.09–8.38) for heart failure, 1.81 (95% CI = 1.29–2.55) for stroke, 2.50 (95% CI = 1.43–4.37) for coronary heart disease, and 2.21 (95% CI = 1.83–2.66) for cardiovascular mortality when compared to women with normotensive pregnancies^51^^,^^52^ These increases in CVD risk are far greater than the risk attributable to conventional cardiovascular risk factors (ie, hypertension, smoking, type 2 diabetes, dyslipidemia).^50^^,^^51^

Outside of the peripartum period, cardiovascular disease is a major contributor to mortality and morbidity for all adults.^53^ Ischemic heart disease provides an example of how both sex and gender are modifiers of disease and treatment. Females are more likely to exhibit coronary microvascular dysfunction, whereas males are more likely to have obstructive coronary artery disease of large vessels.^54^ The result of this differing pathophysiology is that females exhibit higher prevalence of myocardial ischemia without obstructive disease.^54^ In response to acute myocardial infarction, females are more likely to present with symptoms considered “not typical”: interscapular pain, nausea, vomiting, shortness of breath. These symptoms are typical among women but have been defined as atypical because male heart disease and symptoms have constituted the basis of normal.^4^^,^^54^ Women with ischemic heart disease are underdiagnosed, less likely to receive evidence-based treatment and less likely to receive reperfusion when experiencing acute myocardial infarction.^11^^,^^53^^,^^54^ Women are also referred to and attend cardiac rehabilitation in lower numbers than men, with a recent meta-analysis reporting that women are 36% less likely than men to attend a cardiac rehabilitation program.^55^

## Older Adulthood

**Figure 6:**
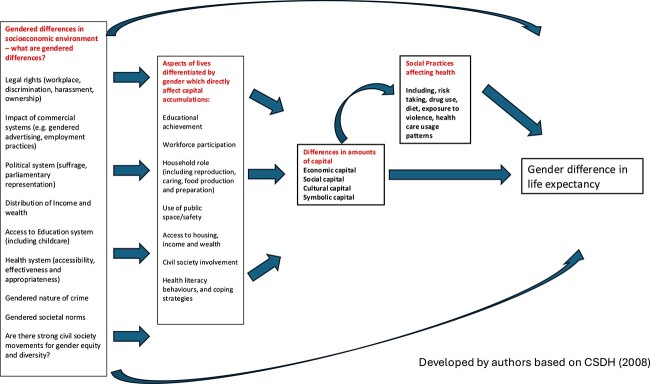
A framework depicting drivers of inequalities and gender differences in life expectancies. Adapted and reprinted with permission from Baum F, Musolino C, Gesesew HA, Popay J. New perspective on why women live longer than men: an exploration of power, gender, social determinants, and capitals. *Public Health*. 2021;18:661. doi: 10.3390/ijerph18020661.

In an investigation of gendered life expectancy differences, Zarulli et al write, “women are the life-expectancy champions.”^23^ Women have a life expectancy that exceeds men’s in every country of the world, in high- and low-resource settings, epidemics, and severe famines.^23^ The explanations for gendered life expectancy differences have challenged researchers because it is equally well established that women report more illness and disability than men, are poorer, and have less control over their lives.^9^^,^^56^ Despite an absolute gap in gendered life expectancy differences, the gap in healthy life expectancy is smaller, meaning that while women live longer, they are more likely to be in poorer health during those years.^23^ Baum and colleagues applied an understanding of economic, cultural, social, and symbolic power to introduce a new framework through which biology and differentials of gender, power, social determinants of health, and capitals influence health outcomes and gendered life expectancy differences (Fig. 6).^56^

Women experience higher stroke-related mortality than men, though this may be in part that women are older when they have a first stroke, and they live longer than men, generally.^57^ Ischemic stroke is most prevalent (84%); rates increase for women at onset of menopause and are more prevalent among women than men >80 years old.^57^^,^^58^ Worse outcomes following stroke are observed in women and are influenced by depression and social isolation.^59^^,^^60^ There is evidence that primary stroke prevention via aspirin therapy is more effective for women, and that acute interventions of thrombolysis and thrombectomy are underutilized in women.^61^^,^^62^ Collectively, this demonstrates how women are differentially at risk of stroke, how they can differentially benefit from preventive measures, and how they are differentially undertreated. It has been reported that males have better rehabilitation outcomes following stroke in studies using functional measure total scores. One recent study found, however, that when scoring items separately, women demonstrate functional recovery as much, if not better than, men, and that outcomes were best among individuals who lived alone prior to their stroke.^63^

Motor performance deteriorates with age, with older women performing worse than older men on nearly all tasks.^64^ The disparity in physical performance over the lifespan may be influenced by women’s differential exposure to occupational and recreational activities that promote physical strength and performance (eg, physical labor, weight training), not simply biological differences between males and females.^6^^,^^40^ Moreover, osteoporosis is a health condition that disproportionately affects older women. Gendered disparities across the life course may influence peak bone mineral density. Examples cited are gendered differences in accepted and promoted PA, gendered social pressures pertaining to body habitus, higher rates of dieting and caloric restriction among women, and social and physical isolation among the elderly.^6^^,^^40^^,^^41^ Such disparities should be considered in prevention and treatment efforts.

Urinary incontinence is another health condition more prevalent among women and one that becomes both more prevalent and severe with age. Among older women, untreated UI and new onset of UI have significant negative consequences that support reconceptualizing treatment and management paradigms. The association between UI and functional impairment is bidirectional, in which women limit activities of daily living, social participation, and health-promoting PA due to fear of UI and this further contributes to decreased strength, balance, and overall physical decline.^65^ The musculoskeletal deconditioning that results can contribute to further loss of pelvic floor muscle performance, negatively affecting continence.^65^^,^^66^ UI is independently associated with mobility impairments among older women, and women with daily UI have over 3 times greater odds of functional dependence.^67^ Impaired mobility and lower body strength are risk factors for developing UI and for increases in UI frequency.^65^^,^^68^^,^^69^ In a study of 673 continent women, 33% (223/673) developed UI in a 4-year time frame.^66^ This group had 1.7 times greater odds of developing sarcopenia and significant declines in physical performance.^66^ Balance and gait impairments are independently associated with urgency UI, with balance and walking speed exhibiting greater impairment with more severe UI.^70^

## Discussion

This study aims to examine the role of sex and gender as key determinants in health outcomes and to highlight factors pertinent to the physical therapy profession. While “female” and “girl and woman” are acknowledged as distinct sex- and gender-based terms and efforts have been made to use the language as it aligns with cited sources, the terms are frequently used interchangeably in the literature and thus, also in this review. Utilizing the Life Course Health Development conceptual framework, research findings were organized according to 5 life phases: (1) maternal and fetal development and influence, (2) infancy and childhood, (3) adolescence and young adulthood, (4) middle-late adulthood, and (5) older adulthood. This research demonstrates that both biological sex and gender identities are determinants of health and may impact access to care and treatment outcomes in physical therapist practice across these life phases. Key findings include the shaping role of gender on motor skills, physical activity, ACL injury and recovery, cardiovascular risks, disease expression, and treatment outcomes, and the disproportionate impact of untreated pelvic floor disorders, including relationships between functional decline, incontinence, and negative social impacts in older adults. The discussion will delve into the implications of these findings for the physical therapist profession, focused on physical therapy research, clinical practice, and education.

Health care professionals and systems, as both “producers of health and health care” and “purveyors of a wider set of societal norms and values are not disconnected and independent from the social, economic, and cultural contexts in which they operate.”^42^^,^^71^ As such, it is recommended that the physical therapy field take a sex- and gender-informed approach aligned with emerging models in medicine.^4^^,^^13^ This means deliberately moving beyond the historic male-centric understanding of health to one that is equitable and accounts for biological and social constructs of sex and gender, respectively, as mediators of health.

## Physical Therapy Research

Broadly, health-related research remains more White, young, and male than the general population.^72^ Elderly, non-White, and pregnant women remain grossly underrepresented in clinical trials, perpetuating disparities in outcomes and practice of evidence-based care.^72^ Despite NIH policies to address sex and gender disparities in research, this has not yielded significant increases in reporting results disaggregated by sex.^8^ There are well-established associations between author gender and sex- and gender-based data analysis, such that papers with a woman as first or senior author are significantly more likely than men to address sex and gender in their analysis.^73^ It appears that the rehabilitation literature reports on participant sex in most papers (>70%); however, the norm is to analyze as a whole. This is demonstrated in the example of Cieza et al, in which sex- and gender-disaggregated data were lacking in the reviews cited supporting LBP treatment.^9^ It is important that physical therapy research considers sex and gender of participants and reports disaggregated outcomes. For example, it is possible that a 40-year-old female employed full-time with a care burden related to family and household responsibilities will be limited in her ability to seek and participate in rehabilitative care compared to a male peer. Evaluating outcomes by sex and gender may allow researchers to elucidate differences between groups that can inform treatment intervention and program design. Beyond disaggregation, there are ample opportunities for researchers to study specific women’s health conditions, such as the potential impact of postpartum cardiac rehabilitation; potential gendered benefits of alternative care models, such as telehealth or hybrid care; or intersectional health outcomes, disparities, and needs, such as women in rural settings or those in the LGBTQ community.

## Physical Therapist Clinical Practice

Most current physical therapist guidelines and protocols are neither sex- nor gender specific. When available evidence demonstrates sex- or gender-based differences in pathophysiology, outcomes, or response to treatment, it is important to develop new or modified guidelines/protocols accordingly. An example of this is the opportunity for developing cardiac rehabilitation programs specific to women’s needs, which may include initiation of cardiac rehabilitation postpartum for those who experienced hypertensive disorders of pregnancy. The lifespan model for understanding pelvic floor function is a useful resource for physical therapists to appreciate their role in supporting pelvic floor function, whether encountering the patient in the immediate context of pregnancy and postpartum, or at a time many years removed. The treatment of UI is another example. Level I evidence supports physical therapist-directed treatment of female UI; however, it remains regarded as a specialty or niche practice, even when prevalence is on par with that of other common musculoskeletal conditions (eg, neck pain, osteoarthritis).^9^ This presents an opportunity for physical therapists to innovate in screening and care delivery and to reconceptualize UI as a health condition that does not lie only with the specialist physical therapist and is not limited to one age group of women. Furthermore, physical therapists are encouraged to apply an enhanced understanding of social and structural determinants of health to understand barriers and opportunities in the context of physical therapist access and engagement. Those involved in pediatric care may adopt language and strategies to mitigate gendered influences on infant fine and gross motor skills. Physical therapists may also lead in health promotion efforts targeting physical activity and fitness among girls during childhood and adolescence. Providing optimal care may require thinking differently about the physical therapist plan of care to account for ways women may be challenged to adequately prioritize their health when they also bear a burden of unpaid care work in the home and community. Alterations in visit frequency and duration, delivery method (remote or telehealth vs in-person), or visit structure, emphasizing self-management strategies in addition to or more than physical therapist-directed exercise and manual therapy may be useful in fostering engagement in rehabilitation and health-promoting activities and favorable treatment outcomes. Physical therapists working with older adults are encouraged to consider the bidirectional relationship between functional impairments and UI, and the related data on alterations in measures of physical health and performance, consideration that may contribute to more comprehensive and effective care. Even from the first physical therapist encounter, clinicians cognizant of sex- and gender-based health impacts across the life course may glean insights about an individual’s health and social history and their present circumstances that enhance the therapeutic relationship and positively influence outcomes. Additionally, physical therapists are encouraged to identify sex- and gender-based disparities in the literature related to their area of focus, as well as to cultivate awareness of one’s own blind spots and biases.

## Physical Therapist Professional Education

Education in health care continues to rely on the 70-kg male as the reference norm for anatomy and physiology, and sex and gender differences are rarely discussed thoroughly in training. For physical therapy to live up to its full scope of practice and doctoral level expectations, sex-based physiology and pathophysiology, and health-related impacts of gender must be introduced at entry-level. The physical therapy community falls short of its stated vision—“Transforming society by optimizing movement to improve the human experience”—if it continues to educate future clinicians, researchers, and professors in a gender-blind manner.^74^ Interest in training in sex- and gender-informed care has been documented and this can permeate all areas of the physical therapy profession.^13^ Sex- and gender-informed care should not be limited to the organizations, academies, and institutions that provide focused training on pelvic health and SRH. Entry-level and post-professional training and all specialty areas of physical therapist practice (eg, pediatrics, geriatrics, orthopedics, neurologic, etc.) can benefit from sex- and gender-informed approaches. Sex and gender health education summits have been described in the medical community and may provide a guide for physical therapist education.^75^ Cross-disciplinary, cross-institutional, and public–private partnerships likely all have a role to play in pursuit of timely progress on sex- and gender-informed physical therapist education.

## Limitations

This work focused on sex and gender disparities specific to female biology and women’s health in the context of physical therapy. We acknowledge the limitation of the sex and gender dichotomy that excludes individuals with intersex biology and those who do not identify as cisgender man or woman. We drew from the literature search selected health conditions that affect women disproportionately and/or differently than men and for which adequate research was available. Existing evidence and the scope of this review limited the number and types of health conditions discussed. Apart from sex and gender disparities between men and boys and women and girls, we recognize that disparities exist within groups of women and girls according to race, ethnicity, ability, socioeconomic status, and other factors. Furthermore, it is important to understand the influences of sex and gender in ever more inclusive and nonbinary ways. A full appraisal of these many determinants of health was beyond the scope of this research, yet where such intersectional data emerged in the search as conducted, it was included in the results. However, this review may inform the sex and gender components of multilevel, multidimensional frameworks of health and disease, where sex and gender intersect with individual-level factors and the physical, social, and political environment to produce health across the lifespan.

## Conclusions

Formal recognition of women’s health in physical therapy in the United States is traced to the 1970s and anchored in SRH. This remains an important area for future research and development, with clear opportunities for growth within the profession. However, it must be recognized that research informing our understanding of physiology, pathophysiology, and response to treatment has been based upon male people or cells or has not been analyzed according to sex. This paper has outlined many ways in which gender- and sex-based differences exist in the health experiences and exposures of women that are germane to physical therapist practice and research and ways we do not know if our treatment outcomes may differ according to sex and gender. The field of sex- and gender-based health care is emerging and being led by the medical and public health communities. Physical therapists play an important role in the health of individuals and communities throughout the lifespan and will benefit from incorporating the knowledge that sex is a biologic modifier of disease and response to treatment, as are gendered influences on clinicians and patients that influence access to health care, care-seeking patterns, use of the health care system, and treatment outcomes. This will allow improved care for and research on behalf of women throughout the physical therapist scope of practice.

## Supplementary Material

PTJ-2023-0594_R1_Supplementary_Material_1_pzae121

PTJ-2023-0594_R1_Supplementary_Material_2_pzae121
