# Supplementary material for: Women’s Health Across the Lifespan: A Sex- and Gender-Focused Perspective
Source: Phys Ther. 2024 Aug 31;104(10):pzae121. doi: 10.1093/ptj/pzae121 (PMC11523629; doi:10.1093/ptj/pzae121)
Supplement: PTJ-2023-0594_R1_Supplementary_Material_2_pzae121 [file ptj-2023-0594_r1_supplementary_material_2_pzae121.pdf]

Supplementary Material 2

Additional works consulted on the topic of Women's Health Across the Lifespan. While not cited in the primary text, the following list represents work that may be of further interest to readers.

Works consulted

1. Schiebinger L, Leopold SS, Miller VM. Editorial policies for sex and gender analysis. *Lancet (London, England)*. 2016;388(10062):2841-2842. doi:10.1016/S0140-6736(16)32392-3
2. Bartz D, Chitnis T, Kaiser UB, et al. Clinical Advances in Sex- and Gender-Informed Medicine to Improve the Health of All: A Review. *JAMA Intern Med*. 2020;180(4):574-583. doi:10.1001/jamainternmed.2019.7194
3. Palabay C, Morales FMR, Zumalacarregui MM, et al. Confronting systemic racism globally. Open Global Rights. Available at: https://www. openglobalrights.org/confronting-systemic-racism-globally/. Accessed March 25, 2021.
4. Halfon N, Forrest CB, Lerner RM, Faustman EM. Handbook of Life Course Health Development; 2018. New York: Springer. doi: 10.1007/978-3-319-47143-3_11.
5. Jones NL, Gilman SE, Cheng TL, et al. Life course approaches to the causes of health disparities. Am J Public Health 2019;109(S1): S48–S55. doi:10.2105/AJPH.2018.304738.
6. Forde AT, Crookes DM, Suglia SF, Demmer RT. The weathering hypothesis as an explanation for racial disparities in health: a systematic review. *Ann Epidemiol*. 2019;33:1-18.e3. doi:10.1016/j.annepidem.2019.02.011
7. Geronimus AT, Hicken M, Keene D, Bound J. "Weathering" and age patterns of allostatic load scores among blacks and whites in the United States. Am J Public Health. 2006 May;96(5):826-33. doi: 10.2105/AJPH.2004.060749. Epub 2005 Dec 27. PMID: 16380565; PMCID: PMC1470581.
8. Crenshaw, Kimberlé W., "On Intersectionality: Essential Writings" (2017). *Faculty Books*. 255. https://scholarship.law.columbia.edu/books/255
9. Hankivsky O. Women's health, men's health, and gender and health: implications of intersectionality. *Soc Sci Med*. 2012;74(11):1712-1720. doi:10.1016/j.socscimed.2011.11.029
10. Boyd RW, Lindo EG, Weeks LD, et al. On racism: a new standard for publishing on racial health inequities. Health Aff Blog 2020;1–9. doi: 10.1377/hblog20200630.939347.
11. Perez CC. *Invisible Women: Exposing Data Bias in a World Designed for Men*. Random House; 2019.
12. Lerner G. *The Creation of Patriarchy (Vol. 1). Women and History; V. 1*. Oxford University Press; 1986.
13. Mayhew R. *The Female in Aristotle's Biology. Reason or Rationalization.* University of Chicago Press; 2004.
14. Radio NP. Russia Lifts Soviet-Era Rules On What Jobs Women Can Do. Published 2021. https://www.npr.org/2021/03/24/980638866/russia-lifts-soviet-era-rules-on-what-jobs-women-could-do#:~:text=Russia Lifts Soviet-Era Rules,Jobs Women Can Do %3A NPR&text=Organization-,Russia Lifts Soviet-Era Rules On What Jobs Women Can,step forward for gen

15. Haslem J. *Therapeutic Management of Incontinence and Pelvic Pain.* 2nd ed. Springer London; 2007.
16. Morris M. *Maternity and Postoperative Exercises*. William Heinemann (Medical Books) Ltd; 1936.
17. Randell M. Training for childbirth from the mother's point of view. In: *Training for Childbirth from the Mother's Point of View*. J&A Churchill Ltd; 1941:58-59.
18. Randell M. Fearless childbirth. In: *Fearless Childbirth*. J & A Churchill Ltd; 1948:28-29; 64-65.
19. Boissonnault J. A historical review of organized women's health physical therapy. *J Assoc Chart Physiother Women's Heal*. 2004;95(95):42-47. http://search.ebscohost.com/login.aspx?direct=true&db=c8h&AN=106456569&site=ehost-live
20. Academy of Pelvic Health Physical Therapy. American Physical Therapy Association. https://aptapelvichealth.org/about/
21. McKinney J. Personal correspondence. 2019.
22. Burton-Jeangros, Claudine Cullati S, Sacker A, Blane D. *"Introduction", A Life Course Perspective on Health Trajectories and Transitions, Springer*.; 2015.
23. Baran Y, Subramaniam M, Biton A, et al. The landscape of genomic imprinting across diverse adult human tissues. *Genome Res*. 2015;25(7):927-936. doi:10.1101/gr.192278.115
24. Zheng Q, Deng Y, Zhong S, Shi Y. Human chorionic gonadotropin, fetal sex and risk of hypertensive disorders of pregnancy: A nested case-control study. *Pregnancy Hypertens*. 2016;6(1):17-21. doi:10.1016/j.preghy.2016.01.006
25. Sykes SD, Pringle KG, Zhou A, Dekker GA, Roberts CT, Lumbers ER. Fetal sex and the circulating renin–angiotensin system during early gestation in women who later develop preeclampsia or gestational hypertension. *J Hum Hypertens*. 2013;28:133-139. doi:10.1038/jhh.2013.51
26. Steier JA, Myking OL, Bergsjø PB. Correlation between fetal sex and human chorionic gonadotropin in peripheral maternal blood and amniotic fluid in second and third trimester normal pregnancies. *Acta Obstet Gynecol Scand*. 1999;78(5):367-371.
27. Verburg PE, Tucker G, Scheil W, Jaap M Erwich JH, Dekker GA, Trelford Roberts C. Sexual Dimorphism in Adverse Pregnancy Outcomes-A Retrospective Australian Population Study. *PLoS One*. 2016;11(7):158807. doi:10.1371/journal.pone.0158807
28. Tan H, Wen SW, Walker M, Fung KFK, Demissie K, Rhoads GG. The association between fetal sex and preterm birth in twin pregnancies. *Obstet Gynecol*. 2004;103(2):327-332. doi:10.1097/01.AOG.0000109427.85586.71
29. Lobue V, Deloache JS. Pretty in pink: The early development of gender-stereotyped colour preferences. *Br J Dev Psychol*. 2011;29(Pt 3):656-667. doi:10.1111/j.2044-835X.2011.02027.x
30. Weisgram ES, Fulcher M, Dinella LM. Pink gives girls permission: Exploring the roles of explicit gender labels and gender-typed colors on preschool children's toy preferences. *J Appl Dev Psychol*. 2014;35(5):401-409. doi:https://doi.org/10.1016/j.appdev.2014.06.004
31. Gromeier M, Koester D, Schack T. Gender Differences in Motor Skills of the Overarm Throw. *Front Psychol*. 2017;8:212. doi:10.3389/fpsyg.2017.00212
32. Ogden CL, Fryar CD, Hales CM, Carroll MD, Aoki Y, Freedman DS. Differences in Obesity Prevalence by Demographics and Urbanization in US Children and Adolescents, 2013-2016. *JAMA*. 2018;319(23):2410-2418. doi:10.1001/jama.2018.5158
33. Fakhouri THI, Hughes JP, Brody DJ, Kit BK, Ogden CL. Physical Activity and Screen-Time

Viewing Among Elementary School-Aged Children in the United States From 2009 to 2010. *JAMA Pediatr*. 2013;167(3):223-229. doi:10.1001/2013.jamapediatrics.122
34. Guseman EH, Tanda R, Haile ZT. Disparities in physical fitness of 6–11-year- old children: the 2012 NHANES National Youth Fitness Survey. *BMC Public Health*. 2020;20(1427):1-8. doi:10.1186/s12889-020-09510-x
35. Cooper C, Westlake S, Harvey N, et al. Review: developmental origins of osteoporotic fracture. *Osteoporos Int*. 2006;17(3):337-347. doi:10.1007/s00198-005-2039-5
36. Thompson WM, Barksdale DJ. Physical inactivity in female African-American adolescents: consequences, costs, & care. *J Natl Black Nurses Assoc*. 2010;21(1):39-45.
37. Kelm J, Ahlhelm F, Anagnostakos K, et al. Gender-specific differences in school sports injuries. *Sport Sport Organ der Gesellschaft fur Orthopadisch-Traumatologische Sport*. 2004;18(4):179-184. doi:10.1055/s-2004-813095
38. Racine M, Tousignant-Laflamme Y, Kloda LA, Dion D, Dupuis G, Choinire M. A systematic literature review of 10 years of research on sex/gender and experimental pain perception - Part 1: Are there really differences between women and men? *Pain*. 2012;153(3):602-618. doi:10.1016/j.pain.2011.11.025
39. Racine M, Tousignant-Laflamme Y, Kloda LA, Dion D, Dupuis G, Choinire M. A systematic literature review of 10 years of research on sex/gender and pain perception - Part 2: Do biopsychosocial factors alter pain sensitivity differently in women and men? *Pain*. 2012;153(3):619-635. doi:10.1016/j.pain.2011.11.026
40. Bartley EJ, Fillingim RB. Sex differences in pain: A brief review of clinical and experimental findings. *Br J Anaesth*. 2013;111(1):52-58. doi:10.1093/bja/aet127
41. Fillingim RB, King CD, Ribeiro-Dasilva MC, Rahim-Williams B, Riley JL. Sex, Gender, and Pain: A Review of Recent Clinical and Experimental Findings. *J Pain*. 2009;10(5):447-485. doi:10.1016/j.jpain.2008.12.001
42. Rogol AD, Roemmich JN, Clark PA. Growth at puberty. *J Adolesc Heal Off Publ Soc Adolesc Med*. 2002;31(6 Suppl):192-200. doi:10.1016/s1054-139x(02)00485-8
43. Papadimitriou A. The Evolution of the Age at Menarche from Prehistorical to Modern Times. *J Pediatr Adolesc Gynecol*. 2016;29(6):527-530. doi:10.1016/j.jpag.2015.12.002
44. Ong KK, Ahmed ML, Dunger DB. Lessons from large population studies on timing and tempo of puberty (secular trends and relation to body size): the European trend. *Mol Cell Endocrinol*. 2006;254-255:8-12. doi:10.1016/j.mce.2006.04.018
45. Walvoord EC. The timing of puberty: is it changing? Does it matter? *J Adolesc Heal Off Publ Soc Adolesc Med*. 2010;47(5):433-439. doi:10.1016/j.jadohealth.2010.05.018
46. Allison CM, Hyde JS. Early menarche: Confluence of biological and contextual factors. *Sex Roles*. 2013;68(1-2):55-64.
47. Mumby HS, Elks CE, Li S, et al. Mendelian Randomisation Study of Childhood BMI and Early Menarche. *J Obes*. 2011;2011. doi:10.1155/2011/180729
48. Krieger N, Kiang M V, Kosheleva A, Waterman PD, Chen JT, Beckfield J. Age at Menarche: 50-Year Socioeconomic Trends Among US-Born Black and White Women. doi:10.2105/AJPH.2014.301936
49. Sangenis P, Drinkwater BL, Loucks AB, Sherman RT, Sundgot-Borgen J, Thompson RA. *Position Stand on THE FEMALE ATHLETE TRIAD: IOC Medical Commission Working Group Women in Sport*.; 2005. http://www.olympic.org/documents/reports/en/en_report_917.pdf

50. Nattiv A, Loucks AB, Manore MM, Sanborn CF, Sundgot-Borgen J, Warren MP. American College of Sports Medicine position stand. The female athlete triad. *Med Sci Sports Exerc*. 2007;39(10):1867-1882. doi:10.1249/mss.0b013e318149f111
51. Mountjoy M, Sundgot-Borgen J, Burke L, et al. The IOC consensus statement: Beyond the Female Athlete Triad-Relative Energy Deficiency in Sport (RED-S). *Br J Sports Med*. 2014;48(7):491-497. doi:10.1136/bjsports-2014-093502
52. Jane De Souza M, Koltun KJ, Etter C V, Southmayd EA. Current Status of the Female Athlete Triad: Update and Future Directions. *Curr Osteoporos Rep*. 2017;15:577-587. doi:10.1007/s11914-017-0412-x
53. Jane De Souza M, Williams NI, Nattiv A, et al. Misunderstanding the Female Athlete Triad: Refuting the IOC Consensus Statement on Relative Energy Deficiency in Sport (RED-S). doi:10.1136/bjsports-2014-093958
54. Feyman Y, Fener NE, Griffith KN. Association of Childcare Facility Closures With Employment Status of US Women vs Men During the COVID-19 Pandemic. Published online 2021. doi:10.1001/jamahealthforum.2021.1297
55. Abdullah B, Ayub SH, Mohd Zahid AZ, Noorneza AR, Isa MR, Ng PY. Urinary incontinence in primigravida: the neglected pregnancy predicament. *Eur J Obstet Gynecol Reprod Biol*. 2016;198:110-115. doi:10.1016/j.ejogrb.2016.01.006
56. Bradley CS, Brown HW, Shippey SS, et al. Generic Health-Related Quality of Life in Patients Seeking Care for Pelvic Organ Prolapse. *Female Pelvic Med Reconstr Surg*. 2021;27(6):337-343. doi:10.1097/SPV.0000000000001069
57. Fontenele MQS, Moreira MA, de Moura ACR, de Figueiredo VB, Driusso P, Nascimento SL. Pelvic floor dysfunction distress is correlated with quality of life, but not with muscle function. *Arch Gynecol Obstet*. 2021;303(1):143-149. doi:10.1007/s00404-020-05770-5
58. Dakic JG, Hay-Smith J, Cook J, Lin K-Y, Calo M, Frawley H. Effect of Pelvic Floor Symptoms on Women's Participation in Exercise: A Mixed-Methods Systematic Review With Meta-analysis. *J Orthop Sports Phys Ther*. 2021;51(7):345-361. doi:10.2519/jospt.2021.10200
59. Bo K, Frawley HC, Haylen BT, et al. An International Urogynecological Association (IUGA)/International Continence Society (ICS) joint report on the terminology for the conservative and nonpharmacological management of female pelvic floor dysfunction. *Neurourol Urodyn*. 2017;36(2):221-244. doi:10.1002/nau.23107
60. Bø K. Physiotherapy management of urinary incontinence in females. *J Physiother*. 2020;66(3):147-154. doi:10.1016/j.jphys.2020.06.011
61. Dumoulin C, Cacciari L, Hay-smith EJC. Pelvic floor muscle training versus no treatment , or inactive control treatments , for urinary incontinence in women (Review). *Cochrane Database Syst Rev*. 2018;(10). doi:10.1002/14651858.CD005654.pub4.www.cochranelibrary.com
62. Favre-Inhofer A, Dewaele P, Millet P, Deffieux X. Systematic review of guidelines for urinary incontinence in women. *J Gynecol Obstet Hum Reprod*. 2020;(2019):101842. doi:10.1016/j.jogoh.2020.101842
63. Lamin E, Parrillo LM, Newman DK, Smith AL. Pelvic Floor Muscle Training: Underutilization in the USA. *Curr Urol Rep*. 2016;17(2):1-7. doi:10.1007/s11934-015-0572-0
64. Minassian VA, Yan X, Lichtenfeld MJ, Sun H, Stewart WF. The Iceberg of Health Care Utilization in Women with Urinary Incontinence. *Int Urogynecol J*. 2012;23(8):1087-1093. doi:10.1007/s00192-012-1743-x.The

65. Waetjen LE, Xing G, Johnson WO, Melnikow J, Gold EB. Factors associated with reasons incontinent midlife women report for not seeking urinary incontinence treatment over 9 years across the menopausal transition. *Menopause*. 2018;25(1):29-37. doi:10.1097/GME.0000000000000943
66. Brown HW, Barnes HC, Lim A, Giles DL, McAchran SE. Better together: multidisciplinary approach improves adherence to pelvic floor physical therapy. *Int Urogynecol J*. 2020;31(5):887-893. doi:10.1007/s00192-019-04090-w
67. Shannon MB, Genereux M, Brincat C, et al. Attendance at Prescribed Pelvic Floor Physical Therapy in a Diverse, Urban Urogynecology Population. *PM R*. 2018;10(6):601-606. doi:10.1016/j.pmrj.2017.11.008
68. Shannon MB, Adams W, Fitzgerald CM, Mueller ER, Brubaker L, Brincat C. Does Patient Education Augment Pelvic Floor Physical Therapy Preparedness and Attendance? A Randomized Controlled Trial. *Female Pelvic Med Reconstr Surg*. 2018;24(2):155-160. doi:10.1097/SPV.0000000000000516
69. Reilly NO, Nelson HD, Conry JM, Frost J, Gregory KD. Annals of Internal Medicine Screening for Urinary Incontinence in Women : A Recommendation From the Women ' s Preventive Services Initiative. Published online 2016. doi:10.7326/M18-0595
70. Auguste T, Gulati M. Optimizing Postpartum Care. *Obstet Gynecol Acog*. 2018;131(5):e140-e150. doi:10.1097/AOG.0000000000002633
71. Wu C, Newman DK, Palmer MH. Unsupervised behavioral and pelvic floor muscle training programs for storage lower urinary tract symptoms in women: a systematic review. *Int Urogynecol J*. 2020;31(12):2485-2497. doi:10.1007/s00192-020-04498-9
72. Dumoulin C, Morin M, Danieli C, et al. Group-Based vs Individual Pelvic Floor Muscle Training to Treat Urinary Incontinence in Older Women: A Randomized Clinical Trial. *JAMA Intern Med*. 2020;180(10):1284-1293. doi:10.1001/jamainternmed.2020.2993
73. Bernard S, Boucher S, McLean L, Moffet H. Mobile technologies for the conservative self-management of urinary incontinence: a systematic scoping review. *Int Urogynecol J*. 2020;31(6):1163-1174. doi:10.1007/s00192-019-04012-w
74. ACOG Practice Bulletin No. 202: Gestational Hypertension and Preeclampsia. *Obstet Gynecol*. 2019;133(1):1. doi:10.1097/AOG.0000000000003018
75. Benschop L, Duvekot JJ, Roeters Van Lennep JE. Future risk of cardiovascular disease risk factors and events in women after a hypertensive disorder of pregnancy. *Heart*. 2019;105:1273-1278. doi:10.1136/heartjnl-2018-313453
76. Grand'Maison S, Pilote L, Schlosser K, Stewart DJ, Okano M, Dayan N. Clinical Features and Outcomes of Acute Coronary Syndrome in Women With Previous Pregnancy Complications. *Can J Cardiol*. 2017;33(12):1683-1692. doi:10.1016/j.cjca.2017.08.025
77. Gad MM, Elgendy IY, Mahmoud AN, et al. Disparities in Cardiovascular Disease Outcomes Among Pregnant and Post-Partum Women. *J Am Heart Assoc*. 2021;10(1):e017832. doi:10.1161/JAHA.120.017832
78. Dayan N, Nerenberg K. Postpartum Cardiovascular Prevention: The Need for a National Health Systems-Based Strategy. *Can J Cardiol*. 2019;35(6):701-704. doi:10.1016/j.cjca.2019.04.004
79. Gladstone RA, Pudwell J, Pal RS, Smith GN. Referral to Cardiology Following Postpartum Cardiovascular Risk Screening at the Maternal Health Clinic in Kingston, Ontario. *Can J Cardiol*. 2019;35(6):761-769. doi:10.1016/j.cjca.2019.03.008

80. Heron M. Deaths: Leading Causes for 2017. *Natl vital Stat reports from Centers Dis Control Prev Natl Cent Heal Stat Natl Vital Stat Syst*. 2019;68(6):1-77.
81. Reis SE, Holubkov R, Conrad Smith AJ, et al. Coronary microvascular dysfunction is highly prevalent in women with chest pain in the absence of coronary artery disease: results from the NHLBI WISE study. *Am Heart J*. 2001;141(5):735-741. doi:10.1067/mhj.2001.114198
82. Dreyer RP, Beltrame JF, Tavella R, et al. Evaluation of gender differences in Door-to-Balloon time in ST-elevation myocardial infarction. *Heart Lung Circ*. 2013;22(10):861-869. doi:10.1016/j.hlc.2013.03.078
83. Mahmoud KD, Gu YL, Nijsten MW, et al. Interhospital transfer due to failed prehospital diagnosis for primary percutaneous coronary intervention: an observational study on incidence, predictors, and clinical impact. *Eur Hear journal Acute Cardiovasc care*. 2013;2(2):166-175. doi:10.1177/2048872613481449
84. Melberg T, Kindervaag B, Rosland J. Gender-specific ambulance priority and delays to primary percutaneous coronary intervention: a consequence of the patients' presentation or the management at the emergency medical communications center? *Am Heart J*. 2013;166(5):839-845. doi:10.1016/j.ahj.2013.07.034
85. D'Onofrio G, Safdar B, Lichtman JH, et al. Sex differences in reperfusion in young patients with ST-segment-elevation myocardial infarction: results from the VIRGO study. *Circulation*. 2015;131(15):1324-1332. doi:10.1161/CIRCULATIONAHA.114.012293
86. Gerdle B, Björk J, Cöster L, Henriksson K, Henriksson C, Bengtsson A. Prevalence of widespread pain and associations with work status: a population study. *BMC Musculoskelet Disord*. 2008;9:102. doi:10.1186/1471-2474-9-102
87. Mogil JS. Sex differences in pain and pain inhibition: multiple explanations of a controversial phenomenon. *Nat Rev Neurosci*. 2012;13(12):859-866. doi:10.1038/nrn3360
88. Smith DG, Rosenstein JE, Nikolov MC. The Different Words We Use to Describe Male and Female Leaders. *Harv Bus Rev*. Published online 2018:1-8. https://hbr.org/2018/05/the-different-words-we-use-to-describe-male-and-female-leaders
89. Murphy H. Picture a Leader. Is She a Woman ? *New York Times*. Published online 2018:1-11. https://www.nytimes.com/2018/03/16/health/women-leadership-workplace.html
90. Abdellatif W, Ding J, Jalal S, et al. Leadership Gender Disparity Within Research-Intensive Medical Schools: A Transcontinental Thematic Analysis. *J Contin Educ Health Prof*. 2019;39(4):243-250. doi:10.1097/CEH.0000000000000270
91. Minehart RD, Foldy EG, Long JA, Weller JM. Challenging gender stereotypes and advancing inclusive leadership in the operating theatre. *Br J Anaesth*. 2020;124(3):e148-e154. doi:10.1016/j.bja.2019.12.015
92. Greenspan JD, Craft RM, LeResche L, et al. Studying sex and gender differences in pain and analgesia: a consensus report. *Pain*. 2007;132 Suppl(Suppl 1):S26-S45. doi:10.1016/j.pain.2007.10.014
93. Krieger N. Genders, sexes, and health: what are the connections--and why does it matter? *Int J Epidemiol*. 2003;32(4):652-657. doi:10.1093/ije/dyg156
94. Nyberg F, Osika I, Evengård B. "The Laundry Bag Project"--unequal distribution of dermatological healthcare resources for male and female psoriatic patients in Sweden. *Int J Dermatol*. 2008;47(2):144-149. doi:10.1111/j.1365-4632.2008.03485.x
95. Tahtinen RM, Cartwright R, Tsui JF, et al. Long-term Impact of Mode of Delivery on Stress

Urinary Incontinence and Urgency Urinary Incontinence: A Systematic Review and Meta-analysis. *Eur Urol*. 2016;70(1):148-158. doi:10.1016/j.eururo.2016.01.037
96. Haider SI, Johnell K, Weitoft GR, Thorslund M, Fastbom J. The influence of educational level on polypharmacy and inappropriate drug use: a register-based study of more than 600,000 older people. *J Am Geriatr Soc*. 2009;57(1):62-69. doi:10.1111/j.1532-5415.2008.02040.x
97. Barford A, Dorling D, Davey Smith G, Shaw M. Life expectancy: women now on top everywhere. *BMJ*. 2006;332(7545):808. doi:10.1136/bmj.332.7545.808
98. Lewer D, Jayatunga W, Aldridge RW, et al. Premature mortality attributable to socioeconomic inequality in England between 2003 and 2018: an observational study. *Lancet Public Heal*. 2020;5(1):e33-e41. doi:10.1016/S2468-2667(19)30219-1
99. Towfighi A, Saver JL, Engelhardt R, Ovbiagele B. A midlife stroke surge among women in the United States. *Neurology*. 2007;69(20):1898-1904. doi:10.1212/01.wnl.0000268491.89956.c2
100. Caso V, Santalucia P, Acciarresi M, Pezzella FR, Paciaroni M. Antiplatelet treatment in primary and secondary stroke prevention in women. *Eur J Intern Med*. 2012;23(7):580-585. doi:10.1016/j.ejim.2012.04.010
101. O'Brien SR, Xue Y. Predicting goal achievement during stroke rehabilitation for Medicare beneficiaries. *Disabil Rehabil*. 2014;36(15):1273-1278. doi:10.3109/09638288.2013.845253
102. Cooper R, Mishra G, Clennell S, Guralnik J, Kuh D. Menopausal status and physical performance in midlife: findings from a British birth cohort study. *Menopause*. 2008;15(6):1079-1085. doi:10.1097/gme.0b013e31816f63a3
103. Salvatore J, Marecek J. Gender in the Gym: Evaluation Concerns as Barriers to Women's Weight Lifting. *Sex Roles*. 2010;63:556-557. doi:10.1007/s11199-010-9800-8
104. Cummings SR, Cawthon PM, Ensrud KE, Cauley JA, Fink HA, Orwoll ES. BMD and risk of hip and nonvertebral fractures in older men: a prospective study and comparison with older women. *J bone Miner Res Off J Am Soc Bone Miner Res*. 2006;21(10):1550-1556. doi:10.1359/jbmr.060708
105. Madill SJ, Pontbriand-Drolet S, Tang A, Dumoulin C. Effects of PFM rehabilitation on PFM function and morphology in older women. *Neurourol Urodyn*. 2013;32(8):1086-1095. doi:10.1002/nau.22370
106. Huang AJ, Brown JS, Thom DH, Fink HA, Yaffe K. Urinary incontinence in older community-dwelling women: the role of cognitive and physical function decline. *Obstet Gynecol*. 2007;109(4):909-916. doi:10.1097/01.AOG.0000258277.01497.4b
107. World Health Organization [WHO]. *World Health Report 2000: Health Systems: Improving Performance.*; 2000. doi:10.1016/S0140-6736(12)61841-8
108. Liu KA, Mager NAD. Women's involvement in clinical trials: historical perspective and future implications. *Pharm Pract (Granada)*. 2016;14(1):708. doi:10.18549/PharmPract.2016.01.708
109. Sugimoto CR, Ahn YY, Smith E, Macaluso B, Larivière V. Factors affecting sex-related reporting in medical research: a cross-disciplinary bibliometric analysis. *Lancet*. 2019;393(10171):550-559. doi:10.1016/S0140-6736(18)32995-7
110. Deshpande BR, Katz JN, Solomon DH, et al. Number of Persons With Symptomatic Knee Osteoarthritis in the US: Impact of Race and Ethnicity, Age, Sex, and Obesity. *Arthritis Care Res (Hoboken)*. 2016;68(12):1743-1750. doi:10.1002/acr.22897

111. Strine TW, Hootman JM. US national prevalence and correlates of low back and neck pain among adults. *Arthritis Rheum*. 2007;57(4):656-665. doi:10.1002/art.22684
